# Maternal Overweight and Obesity and Risks of Severe Birth-Asphyxia-Related Complications in Term Infants: A Population-Based Cohort Study in Sweden

**DOI:** 10.1371/journal.pmed.1001648

**Published:** 2014-05-20

**Authors:** Martina Persson, Stefan Johansson, Eduardo Villamor, Sven Cnattingius

**Affiliations:** 1 Clinical Epidemiological Unit, Department of Medicine Solna, Karolinska University Hospital, Karolinska Institutet, Stockholm, Sweden; 2 Department of Epidemiology, University of Michigan School of Public Health, Ann Arbor, Michigan, United States of America; Hospital Clinic Barcelona, Spain

## Abstract

**Background:**

Maternal overweight and obesity increase risks of pregnancy and delivery complications and neonatal mortality, but the mechanisms are unclear. The objective of the study was to investigate associations between maternal body mass index (BMI) in early pregnancy and severe asphyxia-related outcomes in infants delivered at term (≥37 weeks).

**Methods and Findings:**

A nation-wide Swedish cohort study based on data from the Medical Birth Register included all live singleton term births in Sweden between 1992 and 2010. Logistic regression analyses were used to obtain odds ratios (ORs) with 95% CIs for Apgar scores between 0 and 3 at 5 and 10 minutes, meconium aspiration syndrome, and neonatal seizures, adjusted for maternal height, maternal age, parity, mother's smoking habits, education, country of birth, and year of infant birth. Among 1,764,403 term births, 86% had data on early pregnancy BMI and Apgar scores. There were 1,380 infants who had Apgar score 0–3 at 5 minutes (absolute risk  = 0.8 per 1,000) and 894 had Apgar score 0–3 at 10 minutes (absolute risk  = 0.5 per 1,000). Compared with infants of mothers with normal BMI (18.5–24.9), the adjusted ORs (95% CI) for Apgar scores 0–3 at 10 minutes were as follows: BMI 25–29.9: 1.32 (1.10–1.58); BMI 30–34.9: 1.57 (1.20–2.07); BMI 35–39.9: 1.80 (1.15–2.82); and BMI ≥40: 3.41 (1.91–6.09). The ORs for Apgar scores 0–3 at 5 minutes, meconium aspiration, and neonatal seizures increased similarly with maternal BMI. A study limitation was lack of data on effects of obstetric interventions and neonatal resuscitation efforts.

**Conclusion:**

Risks of severe asphyxia-related outcomes in term infants increase with maternal overweight and obesity. Given the high prevalence of the exposure and the severity of the outcomes studied, the results are of potential public health relevance and should be confirmed in other populations. Prevention of overweight and obesity in women of reproductive age is important to improve perinatal health.

*Please see later in the article for the Editors' Summary*

## Abstract 2

Martina Persson and colleagues use a Swedish national database to investigate the association between maternal body mass index in early pregnancy and severe asphyxia-related outcomes in infants delivered at term.

*Please see later in the article for the Editors' Summary*

## Editors' Summary

**Background:**

Economic, technologic, and lifestyle changes over the past 30 years have created an abundance of cheap, accessible, high-calorie food. Combined with fewer demands for physical activity, this situation has lead to increasing body mass throughout most of the world. Consequently, being overweight or obese is much more common in many high-income and low-and middle-income countries compared to 1980. Worldwide estimates put the percentage of overweight or obese adults as increasing by over 10%, between 1980 and 2008.

As being overweight becomes a global epidemic, its prevalence in women of reproductive age has also increased. Pregnant women who are overweight or obese are a cause for concern because of the possible associated health risks to both the infant and mother. Research is necessary to more clearly define these risks.

**Why Was This Study Done?:**

In this study, the researchers investigated the complications associated with excess maternal weight that could hinder an infant from obtaining enough oxygen during delivery (neonatal asphyxia). All fetuses experience a loss of oxygen during contractions, however, a prolonged loss of oxygen can impact an infant's long-term development. To explore this risk, the researchers relied on a universal scoring system known as the Apgar score. An Apgar score is routinely recorded at one, five, and ten minutes after birth and is calculated from an assessment of heart rate, respiratory effort, and color, along with reflexes and muscle tone. An oxygen deficit during delivery will have an impact on the score. A normal score is in the range of 7–10. Body mass index (BMI) a calculation that uses height and weight, was used to assess the weight status (i.e., normal, overweight, obese) of the mother during pregnancy.

**What Did the Researchers Do and Find?:**

Using the Swedish medical birth registry (a database including nearly all the births occurring in Sweden since 1973) the researchers selected records for single births that took place between 1992 to 2010. The registry also incorporates prenatal care data and researchers further selected for records that included weight and height measurement taken during the first prenatal visit. BMI was calculated using the weight and height measurement. Based on BMI ranges that define weight groups as normal, overweight, and obesity grades I, II, and III, the researchers analyzed and compared the number of low Apgar scoring infants (Apgar 0–3) in each group. Mothers with normal weight gave birth to the majority of infants with Apgar 0–3. In comparison the proportion of low Apgar scores were greater in babies of overweight and obese mothers. The researchers found that the rates of low Apgar scores increased with maternal BMI: the authors found that rates of low Apgar score at 5 minutes increased from 0.4 per 1,000 among infants of underweight women (BMI <18.5) to 2.4 per 1,000 among infants of women with obesity class III (BMI ≥40). Furthermore, overweight (BMI 25.0–29.9) was associated with a 55% increased risk of low Apgar scores at 5 minutes; obesity grade I (BMI 30–34.9) and grade II (BMI 35.0–39.9) with an almost 2-fold and a more than 2-fold increased risk, respectively; and obesity grade ΙΙΙ (BMI ≥40.0) with a more than 3-fold increase in risk. Finally, maternal overweight and obesity also increase the risks for seizures and meconium aspiration in the neonate.

**What Do These Findings Mean?:**

These findings suggest that the risk of experiencing an oxygen deficit increases for the babies of women who are overweight or obese. Given the high prevalence of overweight and obesity in many countries worldwide, these findings are important and suggest that preventing women of reproductive age from becoming overweight or obese is therefore important to the health of their children.

A limitation of this study is the lack of data on the effects of clinical interventions and neonatal resuscitation efforts that may have been performed at the time of birth. Also Apgar scoring is based on five variables and a low score is not the most direct way to determine if the infant has experienced an oxygen deficit. However, these findings suggest that early detection of perinatal asphyxia is particularly relevant among infants of overweight and obese women although more studies are necessary to confirm the results in other populations.

**Additional Information:**

Please access these Web sites via the online version of this summary at http://dx.doi.org/10.1371/journal.pmed.1001648.

- The US National Institutes of Health explains and calculates body mass index
- The NIH also defines the Apgar scoring system
- The United Kingdom's National Health Service has information for pregnant woman who are overweight
- The UK-based Overseas Development Institute discusses how changes in diet have led to a worldwide health crisis in its “Future Diets” publication
- Information about the Swedish health care system is available
- Information in English is available from the National Board of Health and Welfare in Sweden

## Introduction

The obesity epidemic continues to expand globally and WHO projects that 2.3 billion adults will be overweight and 700 million will be obese by 2015 [1]. The prevalence of obesity in pregnancy is high [2]. Maternal overweight and obesity increase the risks of pregnancy and delivery complications [3]–[7], as well as neonatal and infant mortality [3],[8], but the mechanisms underlying these associations are uncertain.

Conventionally, the physical condition of the newborn is assessed using the Apgar scores at 1, 5, and 10 minutes after birth. Low Apgar scores indicate depressed vitality and are a useful tool for prediction of adverse neonatal and long term outcomes [9]–[12]. Although there are a number of possible causes of low Apgar scores [13],[14], among term infants without malformations the vast majority of cases with Apgar scores between 0 and 3 at 5 minutes are due to perinatal asphyxia [15]. Previous studies on offspring of women with overweight and obesity found increased risks of Apgar <7 at 1 or 5 minutes [16]–[18]. It is well recognized that the risk of asphyxia-related complications and long term neurological sequelae is increased in infants with an Apgar score 0–3 compared with an Apgar score 4–6 recorded at 5 or 10 minutes, respectively [11],[19]. Furthermore, the predictive value of a low Apgar score is greater at 10 minutes than a similar Apgar score at 5 minutes [19]. An Apgar score 0–3 at 5 minutes is one of the essential criteria for the presence of perinatal asphyxia as stated by the American Academy of Obstetrics and Gynecology and the American Academy of Pediatrics [20].We have only identified two studies investigating risks of Apgar scores 0–3 at 5 minutes in women with overweight or obesity [21],[22]. In these studies, which included preterm infants and infants with congenital anomalies, maternal obesity was related to low Apgar scores at 5 minutes. Compared with infants of normal weight women, infants of obese mothers are more likely to suffer traumatic delivery, to be large-for-gestational-age (LGA) and in need of neonatal intensive care [6]. Given that LGA infants born at term face an increased risk of infant mortality due to birth asphyxia [23], it is of interest to explore whether maternal overweight and obesity is associated with increased risks of asphyxia-related morbidity in term infants.

We aimed to investigate associations between maternal overweight and obesity and risks of low Apgar scores in a nation-wide cohort study including more than 1.7 million infants born at term in Sweden. We also analyzed maternal overweight and obesity in relation to risks of other severe asphyxia-related conditions; i.e., meconium aspiration and neonatal seizures. We hypothesized that the risks of severe birth asphyxia-related complications would increase with maternal body mass index (BMI).

## Methods

This study was approved by the research ethics committee at Karolinska Institutet, Stockholm, Sweden (number 2012/4:9).This national cohort study was based on data from the Swedish Medical Birth Register (MBR). The registry includes data on more than 98% of all births in Sweden since 1973. The registry is submitted every year to the National Board of Health and Welfare for quality control. In the latest extensive validation it was concluded that coverage and validity of most variables were high [24]. Starting at the first prenatal visit (generally at 8 to 12 weeks of gestation), information is prospectively recorded on maternal socio-demographic factors, maternal and infant anthropometry, and Apgar scores (at 1, 5, and 10 minutes), using standardized antenatal, obstetric, and neonatal forms. Pregnancy, delivery, and neonatal complications are recorded by the woman's and infant's physician, respectively. Diagnoses are classified according the Swedish version of the International Classification of Diseases (ICD). The ninth version (ICD-9) was used from 1992 through 1996, and the tenth version (ICD-10) has been used thereafter. Information is forwarded to the MBR when the mother and infant are discharged from hospital. According to Swedish law we are not able to share the register data used in this study with other researchers.

### Study Population

Between 1992 and 2010, the MBR contains information on 1,926,778 births. After excluding stillbirths (*n = *6,218), multiple births (*n = *56,792), preterm births (before 37 completed gestational weeks, *n = *93,931), records with missing data on gestational age (*n = *2,020), or incomplete maternal identification number (*n = *3,414), the cohort included 1,764,403 live singleton infants delivered at term (≥37 completed weeks). The study population included infants with Apgar scores recorded at 1 and 5 minutes (*n = *1,752,144, corresponding to 99% of the cohort).

### Definition of Outcome

Among infants with complete information on Apgar scores at 1 and 5 minutes (*n = *1,752,144), information on Apgar score at 10 minutes was available in 1,625,210 (93%). In this group with complete information on Apgar scores at all time points there were 1,475,047 infants with an Apgar score of at least 9 at 1 and 5 minutes; Apgar score at 10 minutes was 10 in 1,447,570 infants (98.1%), 4–9 in 25,776 infants (1.7%, of which 25,444 had an Apgar score of 9), and 0–3 in 1,701 infants (0.1%). To minimize the number of infants with missing information on Apgar score at 10 minutes, infants with missing data on Apgar at 10 minutes were given a value of four to ten points if Apgar scores at 1 and 5 minutes were both at least nine. This transformation reduced the number of missing values for Apgar at 10 minutes from 7.2% (*n = *126,934) to 0.58% (*n = *10,191) and decreased the possibility of overestimating rates of low Apgar score at 10 minutes in relation to maternal overweight.

Low Apgar score 5 minutes (0–3), was defined as an Apgar score of 0–3 at 1 and 5 minutes, and an Apgar score of 4–10 at 10 minutes. Low Apgar score at 10 minutes (0–3) was defined as Apgar score of 0–3 at 1, 5, and 10 minutes. Thus, in the group of infants with Apgar scores between 0–3 at 5 minutes, we did not include infants with Apgar scores between 0–3 at 10 minutes. Diagnosis of meconium aspiration was based on ICD-9 code 770.1 and ICD-10 code P.24.0, and diagnosis of neonatal seizures was based on ICD-9 code 779.0 and ICD-10 code P 90.

### Exposure

Maternal weight was measured in light indoor clothes and maternal height was self-reported at the first antenatal visit, occurring within the first trimester in 90% of all pregnancies. BMI was calculated as weight in kilograms divided by the square of the height in meters. Using the WHO criteria, women were categorized as underweight (BMI <18.5), normal weight (BMI 18.5–24.9), overweight (BMI 25–29.9), obesity grade Ι (BMI 30–34.9), obesity grade ΙΙ (BMI 35–39.9), and obesity grade ΙΙΙ (BMI ≥40). In order to explore linear relationships between the exposure and outcome variables, all analyses were also performed with BMI as a continuous variable.

Of all term births (*n = *1,752,144), information on maternal BMI was available in 86% (*n = *1,512,506). Selection bias might occur if the groups of women with and without information on BMI differ from each other. To ascertain whether this might be the case, we investigated a subsample of women with two consecutive singleton births between 1992 and 2010. In the first pregnancy, 435,935 women had information on BMI. In second pregnancy, the distribution of overweight and obesity classes I, II, or III among these women was 25.1%, 7.5%, 2.2%, and 0.7%, respectively. Among women with missing information on BMI in first pregnancy (*n = *75,428), corresponding rates in second pregnancy were 24.5%, 7.2%, 2.1%, and 0.8%, respectively.

### Socio-demographic and Obstetric Covariates

Information on maternal age, height, self-reported parity, and smoking habits was collected at the first prenatal visit. Using each person's unique identification number, information on maternal country of birth and level of education was obtained by individual record linkage between the MBR and the Swedish Population Register and the Education Register, respectively. We identified women with obesity-related complications, including pregestational hypertension (ICD-9 codes 401–405, 642C, 642H and ICD-10 codes I10–I15, O10, O11), pregestational diabetes (ICD-9 codes 250, 648A, and ICD-10 codes E10–E14, O240–0243), gestational diabetes (ICD-9 code 648W, ICD-10 code O244), and preeclampsia (ICD-9 codes 642E–642G and ICD-10 codes O14, O15). We also identified infants with any congenital anomalies (ICD-9 codes 744–759 and ICD-10 codes Q00–Q99), but from this group we excluded infants with minor malformations, including undescended testicle, preauricular appendage, congenital nevus, and hip dislocation. Information on gestational age was generally based on an early ultrasonic scan, offered to all pregnant women in Sweden. Ninety-five percent of the women accept this offer, and the scan is generally performed at 16 to 18 weeks of gestation [25]. When information from an ultrasonic scan was not available, gestational age was estimated based on date of last menstrual period.

### Statistical Analyses

Rates of Apgar scores 0–3 at 5 and 10 minutes, meconium aspiration, and neonatal seizures were calculated as the proportion of infants with these outcomes in the study population. Logistic regression analyses were performed to estimate the risk of neonatal complications in different maternal BMI categories (underweight, overweight, and obesity grade Ι–ΙΙΙ) as compared with normal-weight women. The analyses were also performed with BMI as a continuous variable. In all analyses, the generalized estimating equation method was applied to correct for repeated pregnancies, using the GENMODE procedure. In the multivariable analyses, estimates were adjusted for maternal height, age, parity, smoking in early pregnancy, level of education, mother's country of birth, and year of infant birth. These variables were categorized and entered in the model as listed in Table 1. In a second multivariate model, odds ratios were also adjusted for mode of delivery. To explore potential contributions of maternal obesity-related diseases and congenital malformations, all analyses were repeated after excluding infants of women with chronic hypertension, preeclampsia, pregestational or gestational diabetes, and infants with malformations. All analyses were performed using SAS software package version 9.3 (SAS Institute, Inc.).

**Table 1:** Maternal characteristics and rates of low Apgar scores at 5 and 10 minutes in live singleton term births in Sweden 1992–2010.

Maternal Characteristics	Number of Infants	Apgar Score 0–3 at 5 min	Apgar Score 0–3 at 10 min
		Number (Rate/1,000)	Number (Rate/1,000)
**Total**	1,752,144	1,380 (0.8)	894 (0.5)
**BMI**			
<18.5	38,516	17 (0.4)	16 (0.4)
18.5 to <25	961,710	604 (0.6)	431 (0.4)
25 to <30	364,524	353 (1.0)	212 (0.6)
30 to <35	107,404	122 (1.1)	66 (0.6)
35 to <40	30,365	46 (1.5)	21 (0.7)
≥40	9,987	24 (2.4)	12 (1.2)
Data missing	239,638	214 (0.9)	136 (0.6)
**Height (cm)**			
<155	50,206	67 (1.3)	25 (0.5)
155–164	571,095	542 (1.0)	316 (0.6)
165–174	845,894	577 (0.7)	414 (0.5)
≥175	156,750	81 (0.5)	65 (0.4)
Data missing	128,199	113 (0.9)	74 (0.6)
**Age (years)**			
−19	33,354	25 (0.8)	19 (0.6)
20–24	263,386	190 (0.7)	133 (0.5)
25–29	582,884	430 (0.7)	265 (0.4)
30–34	571,162	455 (0.8)	311 (0.5)
≥35	301,358	280 (0.9)	166 (0.6)
**Parity**			
1	749,517	801 (1.1)	459 (0.6)
2	645,987	350 (0.5)	282 (0.4)
3	248,947	150 (0.6)	103 (0.4)
≥4	107,693	79 (0.7)	50 (0.5)
**Cigarette smoking**			
No	1,462,284	1,137 (0.8)	725 (0.5)
Smoker	199,926	160 (0.8)	117 (0.6)
Data missing	88,637	83 (0.9)	52 (0.6)
**Education (years)**			
≤9	173,405	152 (0.9)	83 (0.5)
10–11	358,029	299 (0.8)	187 (0.5)
12	421,061	364 (0.9)	214 (0.5)
13–14	258,242	204 (0.8)	128 (0.5)
≥15	510,432	332 (0.6)	263 (0.5)
Data missing	30,975	29 (0.9)	19 (0.6)
**Mother's country of birth**			
Sweden	1,418,953	1,095 (0.8)	711 (0.5)
Nordic	40,636	20 (0.5)	21 (0.5)
Non-Nordic	274,507	249 (0.9)	144 (0.5)
Data missing	18,048	16 (0.9)	18 (1.0)
**Hypertensive disease**			
No	1,702,434	1,294 (0.8)	865 (0.5)
Chronic hypertension	9,971	17 (1.7)	6 (0.6)
Preeclampsia	39,739	69 (1.7)	23 (0.6
**Diabetic disease**			
No	1,730,187	1,336 (0.8)	878 (0.5)
Gestational diabetes	15,636	29 (1.8)	11 (0.7)
Pregestational diabetes	6,321	15 (2.4)	5 (0.8)
**Year of birth**			
1992–1996	497,302	341 (0.7)	292 (0.6)
1997–2000	317,401	262 (0.8)	232 (0.7)
2001–2005	442,371	370 (0.8)	151 (0.3)
2006–2010	495,070	407 (0.8)	219 (0.4)

## Results

The total number of infants who had a low Apgar score (0–3) at 5 minutes and at 10 minutes were 1,380 (absolute risk 0.8 per 1,000) and 894 (absolute risk 0.5 per 1,000), respectively (Table 1). The majority of infants with Apgar 0–3 were born to mothers with normal weight. Rates of low Apgar score at 5 and 10 minutes increased with maternal BMI. Rates of low Apgar score at 5 minutes increased from 0.4 per 1,000 among infants of underweight women (BMI <18.5) to 2.4 per 1,000 among infants of women with obesity class III (BMI ≥40). The rates of low Apgar scores were increased in infants of mothers who were older (≥35 years), primiparous, had chronic hypertension, preeclampsia, pregestational or gestational diabetes. Rates of low Apgar scores at 5 minutes did not differ between smokers and non-smokers and rates at 10 minutes were only slightly higher in smokers. Rates of low Apgar scores at 5 minutes were lower in underweight women (0.4%) as compared with women of normal weight (0.6%). Of all infants with Apgar 0–3 at 5 minutes, 298 of 1,380 (17%) also had a diagnosis of meconium aspiration and/or neonatal seizures. Among infants with Apgar 0–3 at 10 minutes, the corresponding number and proportion of infants with meconium aspiration and/or neonatal seizures were 167 of 894 (19%).

The risks of low Apgar scores increased with maternal BMI (Table 2). Compared with infants of women of normal weight (BMI 18.5–24.9), the risks of low Apgar scores increased with maternal BMI (Table 2),demonstrating a linear relationship between the exposure and outcome. Overweight (BMI 25.0–29.9) was associated with a 55% increased risk of low Apgar scores at 5 minutes; obesity grade I (BMI 30–34.9) and grade II (BMI 35.0–39.9) with an almost 2-fold and a more than 2-fold increased risk, respectively; and obesity grade ΙΙΙ (BMI ≥40.0) with a more than 3-fold increase in risk (Table 2, adjusted model 1). Corresponding increments in risks of low Apgar scores at 10 minutes were only slightly lower. Overweight was associated with a 32% increased risk of low Apgar scores at 10 minutes, obesity grade I (BMI 30–34.9) and obesity grade II (BMI 35.0–39.9) with more than 50 and 80% increments in risk, respectively. Obesity grade III (BMI ≥40) was associated with a three and a half times increased risk (Table 2, adjusted model 1). Excluding women with obesity-related diseases (chronic hypertension, preeclampsia, pregestational or gestational diabetes) and infants with malformations from the analyses did not substantially change these risk estimates (Table 2, adjusted models 2).

**Table 2:** Maternal body-mass index and odds ratios for low Apgar scores at 5 and 10 minutes: live singleton term births in Sweden 1992–2010.

BMI Category	Apgar Score 0–3 at 5 min			Apgar Score 0–3 at 10 min		
	OR (95% CI)			OR (95% CI)		
	Crude	Adjusted^a^		Crude	Adjusted^a^	
		Model 1	Model 2^b^		Model 1	Model 2^b^
<18.5	0.70 (0.43–1.14)	0.69 (0.42–1.14)	0.67 (0.40–1.15)	0.93 (0.56–1.53)	0.95 (0.56–1.59)	0.87 (0.50–1.52)
18.5 to <25 (ref)	1.00	1.00	1.00	1.00	1.00	1.00
25 to <30	1.54 (1.35–1.76)	1.55 (1.35–1.77)	1.55 (1.34–1.79)	1.30 (1.10–1.53)	1.32 (1.10–1.58)	1.40 (1.17–1.68)
30 to <35	1.81 (1.49–2.20)	1.84 (1.51–2.25)	1.94 (1.57–2.41)	1.37 (1.05–1.78)	1.57 (1.20–2.07)	1.61 (1.21–2.14)
35 to <40	2.41 (1.79–3.26)	2.38 (1.75–3.24)	2.09 (1.44–3.02)	1.54 (1.00–2.39)	1.80 (1.15–2.82)	1.99 (1.24–3.21)
≥40	3.83 (2.55–5.77)	3.58 (2.33–5.51)	3.29 (1.96–5.53)	2.68 (1.51–4.76)	3.41 (1.91–6.09)	4.06 (2.23–7.41)
Per unit increase	1.07 (1.06–1.08)	1.07 (1.05–1.08)	1.06 (1.05–1.08)	1.04 (1.02–1.05)	1.05 (1.03–1.06)	1.06 (1.04–1.07)

a Both models are adjusted for maternal country of birth, smoking in early pregnancy, education, parity, height, maternal age, and year of infant birth. Number of births included in the analysis was for Apgar score 0–3 at 5 minutes: crude model *n = *1,512,506 births, model 1 *n = *1,462,187 births, and model 2 *n = *1,374,148 births. For Apgar score 0–3 at 10 minutes, the corresponding numbers were *n = *1,504,050; *n = *1,451,501; and *n = *1,366,720, respectively.

b Women with any type of diabetes, chronic hypertension, preeclampsia, or having infants with malformations are excluded.

The risks of meconium aspiration and neonatal seizures also increased with maternal BMI (Table 3). Compared with infants of normal weight women, there was approximately a 50% risk increase of meconium aspiration and neonatal seizures in infants of overweight mothers, and corresponding risks were almost doubled in infants of mothers with obesity grade Ι. Infants of mothers with obesity grade II or ΙΙΙ faced a tripled risk of meconium aspiration. In addition, obesity grades II and III were related to doubled and quadrupled risk of neonatal seizures, respectively (Table 3, adjusted models 1). Repeating the analyses in a subsample of women without obesity-related diseases and with infants without malformations did not change these results (Table 3, adjusted model 2). Finally we investigated whether mode of delivery influenced the associations between maternal BMI and risks of low Apgar scores (Table 4), meconium aspiration, and neonatal seizures (Table 5). However, when we also adjusted for mode of delivery, the BMI related risks were only slightly attenuated (Tables 4 and 5).

**Table 3:** Maternal body-mass index and odds ratios for meconium aspiration and neonatal seizures: live singleton term births in Sweden 1992–2010.

BMI Category	Meconium Aspiration			Neonatal Seizures		
	OR (95% CI)			OR (95% CI)		
	Crude	Adjusted^a^		Crude	Adjusted^a^	
		Model 1	Model 2^b^		Model 1	Model 2^b^
<18.5	0.78 (0.56–1.10)	0.78 (0.55–1.10)	0.78 (0.55–1.11)	0.72 (0.51–1.02)	0.77 (0.54–1.09)	0.79 (0.55–1.14)
18.5 to <25 (ref)	1.00	1.00	1.00	1.00	1.00	1.00
25 to <30	1.45 (1.31–1.60)	1.55 (1.41–1.72)	1.58 (1.43–1.76)	1.40 (1.27–1.54)	1.45 (1.31–1.61)	1.47 (1.32–1.64)
30 to <35	1.65 (1.42–1.91)	1.90 (1.63–2.22)	1.90 (1.61–2.23)	1.67 (1.44–1.93)	1.79 (1.54–2.09)	1.72 (1.46–2.04)
35 to <40	2.59 (2.09–3.20)	2.98 (2.39–3.71)	2.88 (2.25–3.68)	2.05 (1.62–2.59)	2.20 (1.73–2.79)	1.88 (1.41–2.51)
≥40	2.42 (1.66–3.52)	2.95 (2.03–4.31)	3.09 (2.04–4.68)	3.79 (2.78–5.18)	4.04 (2.94–5.55)	4.15 (2.91–5.91)
Per unit increase	1.06 (1.05–1.07)	1.07 (1.06–1.08)	1.07 (1.06–1.08)	1.06 (1.05–1.06)	1.06 (1.05–1.07)	1.06 (1.05–1.07)

a Both models are adjusted for maternal country of birth, smoking in early pregnancy, education, parity, height, maternal age, and infant year of birth. Number of births included in the analyses of meconium aspiration and neonatal seizures: crude models *n = *1,512,506 births, model 1 *n = *1,462,187 births, and model 2 *n = *1,374,148 births.

b Women with any type of diabetes, chronic hypertension, preeclampsia, or having infants with malformations are excluded.

**Table 4:** Maternal body-mass index and odds ratios for low Apgar scores at 5 and 10 minutes: live singleton term births in Sweden 1992–2010.

BMI Category	Apgar Score 0–3 at 5 min			Apgar Score 0–3 at 10 min		
	OR (95% CI)			OR (95% CI)		
	Crude	Adjusted		Crude	Adjusted	
		Model 1	Model 2		Model 1	Model 2
<18.5	0.70 (0.43–1.14)	0.76 (0.46–1.25)	0.74 (0.43–1.25)	0.93 (0.56–1.53)	0.98 (0.58–1.65)	0.91 (0.52–1.59)
18.5 to <25 (ref)	1.00	1.00	1.00	1.00	1.00	1.00
25 to <30	1.54 (1.35–1.76)	1.39 (1.21–1.60)	1.39 (1.20–1.61)	1.30 (1.10–1.53)	1.21 (1.01–1.45)	1.28 (1.07–1.54)
30 to <35	1.81 (1.49–2.20)	1.52 (1.24–1.86)	1.60 (1.28–1.98)	1.37 (1.05–1.78)	1.40 (1.07–1.84)	1.42 (1.06–1.89)
35 to <40	2.41 (1.79–3.26)	1.84 (1.34–2.51)	1.61 (1.11–2.34)	1.54 (1.00–2.39)	1.53 (0.97–2.40)	1.68 (1.04–2.72)
≥40	3.83 (2.55–5.77)	2.62 (1.70–4.04)	2.40 (1.42–4.04)	2.68 (1.51–4.76)	2.81 (1.57–5.03)	3.30 (1.80–6.03)
Per unit increase	1.07 (1.06–1.08)	1.05 (1.04–1.06)	1.05 (1.03–1.06)	1.04 (1.02–1.05)	1.04 (1.02–1.05)	1.04 (1.02–1.06)

In addition to regression models used in Table 3, odds ratios are further adjusted for mode of delivery.

**Table 5:** Maternal body-mass index and odds ratios for meconium aspiration and neonatal seizures; live singleton term births in Sweden 1992–2010.

BMI Category	Meconium Aspiration			Neonatal Seizures		
	OR (95% CI)			OR (95% CI)		
	Crude	Adjusted		Crude	Adjusted	
		Model 1	Model 2		Model 1	Model 2
<18.5	0.78 (0.56–1.10)	0.83 (0.59–1.17)	0.84 (0.59–1.20)	0.72 (0.51–1.02)	0.77 (0.54–1.10)	0.80 (0.55–1.15)
18.5 to <25 (ref)	1.00	1.00	1.00	1.00	1.00	1.00
25 to <30	1.45 (1.31–1.60)	1.41 (1.28–1.57)	1.45 (1.31–1.61)	1.40 (1.27–1.54)	1.38 (1.25–1.53)	1.40 (1.26–1.56)
30 to <35	1.65 (1.42–1.91)	1.63 (1.39–1.90)	1.62 (1.37–1.91)	1.67 (1.44–1.93)	1.62 (1.39–1.89)	1.56 (1.31–1.85)
35 to <40	2.59 (2.09–3.20)	2.40 (1.92–3.00)	2.35 (1.83–3.01)	2.05 (1.62–2.59)	1.96 (1.54–2.49)	1.69 (1.26–2.25)
≥40	2.42 (1.66–3.52)	2.32 (1.59–3.39)	2.45 (1.62–3.72)	3.79 (2.78–5.18)	3.50 (2.54–4.82)	3.61 (2.53–5.16)
Per unit increase	1.06 (1.05–1.07)	1.06 (1.05–1.06)	1.06 (1.05–1.07)	1.06 (1.05–1.06)	1.05 (1.04–1.06)	1.05 (1.04–1.06)

In addition to regression models used in Table 3, odds ratios are further adjusted for mode of delivery.

## Discussion

This population-based cohort study of more than 1.7 million births clearly demonstrates that maternal overweight and obesity is associated with increased risks of low Apgar scores (0–3) at 5 and 10 minutes, meconium aspiration, and neonatal seizures in term infants. The risks of all outcomes increased with maternal BMI category in a dose-response pattern.

To our knowledge, this is the first study to investigate risks of severe asphyxia-related complications in relation to maternal BMI in a cohort of term infants without malformations. Obesity-related diseases that may increase the risks of low Apgar scores, fetal hypoxia, and related complications include preeclampsia and diabetes [26],[27]. Maternal obesity also increases risks of some congenital anomalies [28], which are associated with increased risks of asphyxia-related neonatal complications [14]. However, excluding infants born to mothers with diabetes or preeclampsia and infants with congenital anomalies did not substantially change the risks associated with overweight/obesity, suggesting that high maternal BMI has an independent negative impact on birth asphyxia.

The increased risks of perinatal asphyxia-related complications in term infants of obese mothers may partly be attributed to shoulder dystocia [15] and otherwise traumatic labor due to fetal macrosomia, both conditions being more frequently seen in obese women [6]. Fetal hyperinsulinemia is another possible contributing factor to the increased risks of birth asphyxia in offspring of obese mothers. In offspring of obese mothers without diabetes, a strong association between increasing maternal BMI and fetal hyperinsulinemia was recently demonstrated [7]. Maternal obesity is associated with insulin resistance [29], which enhances nutrient transfer across the placenta and induces fetal hyperinsulinemia, which in turn may lead to chronic fetal hypoxia.

A strong correlation (r^2^ = 0.84) has been reported between BMI in early pregnancy and fat mass [30]. Total fat mass, including visceral fat mass, increases in pregnancy, especially in overweight and obese women [31]. Visceral fat mass is associated with insulin resistance, inflammation [32], and increased levels of non-esterified fatty acids in the circulation, which may lead to lipotoxicity [33]. Lipotoxicity in turn induces oxidative stress and endothelial dysfunction in both maternal and placental tissues with decreased trophoblast invasion and altered placental metabolism [33]. Obesity is also associated with an increased risk of thrombosis during pregnancy [34] and a state of inflammation in the placenta [35], both of which may reduce placental blood flow. It is possible that altered metabolism, inflammation, and endothelial dysregulation in placental tissues may contribute to the increased risk of birth asphyxia in offspring of obese women. It has also been demonstrated that pregnancies complicated by obesity are associated with increased risk of cord coiling, a risk factor for fetal distress [36], which may lead to birth asphyxia.

The strengths of the present study include the large number of births, enabling analyses of risks in relation to a large range of BMI values above the normal, also including the most severe forms of obesity. The population-based study design, with prospectively collected data, limits the risks of selection and information bias. Finally, we were able to adjust for potential key confounders.

Some study limitations should be noted. Perinatal asphyxia is commonly defined as the presence of arterial cord pH≤7.0 and Apgar scores 0–3 at 5 minutes. In the absence of data on arterial cord pH, we used Apgar scores 0–3 at 5 and 10 minutes as asphyxia-related outcomes. Low Apgar scores at 5 and 10 minutes are associated with increased risks of long term neurological sequelae [9],[11],[12]. In term infants, a low Apgar score (0–3) at 5 minutes is associated with an 8-times higher risk of neonatal mortality than cord blood acidosis [10]. Other causes of low Apgar scores, besides birth asphyxia, include preterm delivery and congenital conditions and malformations [14]. However, by only including term infants and excluding infants with malformations in the analyses, we have likely reduced the number of infants with low Apgar scores for reasons other than birth asphyxia. We lack information about obstetric and neonatal interventions, potentially influencing Apgar scores and neonatal morbidity.

Overweight women of reproductive age tend to slightly over-report their height and under-report their weight. In the present study, BMI was calculated from self-reported height but on measured weight, which is an advantage over self-reported weight. Self-reporting errors in height may, if anything, have led to an underestimation of risks associated with maternal overweight and obesity. Information on early pregnancy BMI was missing in 14% of mothers giving birth to singleton infants at term. We believe that the possibility of selection bias due to missing data on BMI was small as women with two pregnancies in the dataset had the same distribution of BMI in the second pregnancy, regardless of having BMI recorded or not in the first pregnancy. Furthermore, in offspring of women with missing data on pregnancy BMI, rates of low Apgar scores at 5 and 10 minutes were in the same range as for offspring of normal or overweight women. As information on gestational weight gain was only available in a subset of the population, weight gain was not included in the analyses. However, in a recent Cochrane review it was concluded that abnormal weight gain in pregnancy was associated with increased risks for abnormal fetal growth and preterm delivery, whereas evidence for a negative impact on risks of other neonatal complications was weak [37]. A Swedish study demonstrated that abnormal weight gain during pregnancy (<8 kg or >16 kg) did not significantly alter the risk of low Apgar scores in offspring of women with overweight and different degrees of maternal obesity [17].

All pregnant women in Sweden are screened for gestational diabetes, based on repeated random urine and capillary plasma glucose. In spite of this, we cannot exclude that there were undiagnosed cases of gestational diabetes. However, excluding women with known pregestational and gestational diabetes from the analyses did not reduce obesity-related risks of asphyxia-associated complications. Information about maternal smoking was based on self-report at the first visit to antenatal care, and the validity of self-reported smoking in early pregnancy in Sweden is acceptable [38].

In summary, this population-based cohort study from Sweden clearly demonstrates increased risks of perinatal asphyxia-related complications with increasing maternal BMI in infants delivered at term. Given the high prevalence of maternal overweight and obesity in many countries and the severity of the outcomes studied, the results are of potential public health relevance and should be confirmed in other populations. Our results suggest that early detection of perinatal asphyxia is particularly relevant among infants of obese women. Prevention of overweight and obesity in women of reproductive age is an important strategy to improve perinatal health.

## Abbreviations

BMI: body mass index
OR: odds ratio
